# Neuromagnetic responses to tactile stimulation of the fingers: Evidence for reduced cortical inhibition for children with Autism Spectrum Disorder and children with epilepsy

**DOI:** 10.1016/j.nicl.2017.06.026

**Published:** 2017-06-23

**Authors:** William Gaetz, Michael T. Jurkiewicz, Sudha Kilaru Kessler, Lisa Blaskey, Erin S. Schwartz, Timothy P.L. Roberts

**Affiliations:** a Lurie Family Foundations MEG Imaging Center, Department of Radiology, Children's Hospital of Philadelphia, United States; b Department of Radiology, Children’s Hospital of Philadelphia and Perelman School of Medicine, University of Pennsylvania, United States; c Department of Neurology, Children's Hospital of Philadelphia and Perelman School of Medicine, University of Pennsylvania, United States; d Children's Hospital of Philadelphia, Department of Radiology and Center for Autism Research, United States; e Department of Pediatrics, Children's Hospital of Philadelphia and Perelman School of Medicine, University of Pennsylvania, United States

**Keywords:** Autism Spectrum Disorder, Epilepsy, Magnetoencephalography (MEG), Post-excitatory inhibition, Somatosensory evoked fields (SEFS), Tactile stimulation

## Abstract

The purpose of this study was to compare somatosensory responses from a group of children with epilepsy and a group of children with autism spectrum disorder (ASD), with age matched TD controls. We hypothesized that the magnitude of the tactile “P50m” somatosensory response would be reduced in both patient groups, possibly due to reduced GABAergic signaling as has been implicated in a variety of previous animal models and in vivo human MRS studies. We observed significant (~ 25%) decreases in tactile P50m dipole moment values from the source localized tactile P50m response, both for children with epilepsy and for children with ASD. In addition, the latency of the tactile P50m peak was observed to be equivalent between TD and ASD groups but was significantly delayed in children with epilepsy by ~ 6 ms. Our data support the hypothesis of impaired GABAergic signaling in both children with ASD and children with epilepsy. Further work is needed to replicate these findings and directly relate them to both in vivo measures of GABA via e.g. magnetic resonance spectroscopy and psychophysical assessments of somatosensory function, and behavioral indices.

## Highlights

• We observed decreased magnitudes of the somatosensory response for both children with epilepsy and for children with autism.
• The latency of the peak response was equivalent between TD and autism groups but delayed in children with epilepsy by ~ 6 ms.
• Our data support the hypothesis of impaired GABAergic signaling in both children with autism and children with epilepsy.

## 1 Introduction

The recording of somatosensory evoked potentials (SEPs) and evoked magnetic fields (SEFs) has been common practice for the assessment of the functional integrity of the human somatosensory system (Anziska and Cracco, 1983, Cracco et al., 1982, Hari and Forss, 1999, Hari et al., 1984, Kakigi et al., 2000). Clinically, SEP/SEF applications are varied, including the measurement of nerve conduction velocity abnormalities in the setting of white matter lesions (e.g., evoked-response latencies are known to be delayed in multiple sclerosis) (Walsh et al., 1982), identifying the central sulcus in patients in need of pre-surgical functional mapping (Sheth et al., 2013), and to monitor the integrity of afferent somatosensory pathways (Seyal and Mull, 2002). SEPs/SEFs are typically elicited by electrical stimuli applied to a peripheral nerve, such as stimulation of the median nerve at the wrist. Electrical median nerve stimulation produces strongly synchronized afferent responses by simultaneously activating a large number of mixed (i.e., both sensory and motor) nerve fibers governing the palmar thumb, index and middle fingers, and with contributions from a mixture of different kinds of afferent fibers (e.g., cutaneous mechanoreceptors, joint, and muscle spindle afferents). Median nerve stimulation produces a sequence of deflections in the averaged evoked response, whose neural origins have been localized to primary somatosensory cortex (SI) using equivalent current dipole (ECD) modeling (Hari et al., 1984, Allison et al., 1991, Allison et al., 1989, Baumgartner et al., 1993, Mauguiere et al., 1997, Lin et al., 2005, Huttunen and Lauronen, 2012). The first cortical response following median nerve stimulation peaks at about 20 milliseconds (ms) and has been termed the “_MN_N20” using electroencephalography (EEG), or “_MN_N20m” using magnetoencephalography (MEG). ECD source orientation of the _MN_N20m component is typically posterior-anterior, indicating current flow from deep to superficial cortical layers (Huttunen and Lauronen, 2012, Nevalainen et al., 2014). The _MN_N20m component is followed by a _MN_P35m component which has been localized to approximately the same cortical area, but with the opposite anterior-posterior dipole orientation, indicating current flow from superficial to deep cortical layers of Brodmann area 3b (Lin et al., 2005, Huttunen and Lauronen, 2012, Nevalainen et al., 2014).

Electrical median nerve stimulation can be unpleasant, even painful, thus challenging for use with clinical pediatric populations. Alternatively, a variety of more natural tactile stimulation methods have been developed for studying the somatosensory responses: tactile tapping (Pihko et al., 2009), brushing (Cheyne et al., 2003, Gaetz and Cheyne, 2006), vibration (Nangini et al., 2006), air puff (Matsumiya and Mostofsky, 1972, Schieppati and Ducati, 1984, Forss et al., 1994) as well passive movement (Xiang et al., 1997, Bourguignon et al., 2014). Tactile stimuli can be applied to any discrete area of the skin (e.g., fingers, lips, toes, etc.) and as such, these responses have also been used to detail the somatotopic representations of the human somatosensory cortex non-invasively with MEG (Jamali and Ross, 2013, Inoue et al., 2013). Not surprisingly, transient mechanical stimulation of an individual fingertip, for example, tends to evoke similar response waveforms as median nerve stimulation. However, due to relatively slower nerve conduction velocities, responses to transient mechanical stimulation occur with slightly longer evoked-response latencies than those produced using median nerve stimulation (Nevalainen et al., 2014). Like the _MN_N20m/P35m complex produced by electrical stimulation, mechanical stimulation of a fingertip produces a tactile N30m/P50m complex which likely also activates neurons in the posterior bank of the post central sulcus including area 3b; however, such stimulation may also include other areas such as somatosensory areas 3a and 1 which may also possibly contribute to tactile SEFs_._ As expected, ECD analysis of this first tactile N30m response results in a forward pointing dipole localized to primary somatosensory cortex, and is thus likely the tactile analog of the _MN_N20m (Huttunen and Lauronen, 2012, Nevalainen et al., 2014). Similarly, the tactile P50m ECD source is oriented anterior-posteriorly and thus likely represents the tactile analog of the _MN_P35m (Nevalainen et al., 2014).

Considerable evidence suggests the first cortical somatosensory response for both the _MN_N20m and tactile N30m arises from glutamatergic excitatory post-synaptic potentials (EPSPs) from depolarized pyramidal neurons in somatosensory area 3b (Hari et al., 1984, Allison et al., 1989, Ikeda et al., 2005, Wood et al., 1985, Baumgartner et al., 1991). Although much less is known about the post-excitatory _MN_P35m or tactile P50m components, converging evidence now suggests that the second SEF component following a transient stimulus represents *post-excitatory inhibition* from a distinct neuronal population in sensorimotor cortices due to inhibitory post-synaptic potentials (IPSPs) typically governed by gamma-amino butyric acid (GABA) inhibitory signaling (Egawa et al., 2008, Deisz and Prince, 1989, Wikstrom et al., 1996, Beierlein et al., 2003, Reyes et al., 1998, Amiet et al., 2008). The most direct support for this position stems from the observation that the _MN_P35m component is absent in patients with the neurodevelopmental disorder Angelman syndrome, a disorder most commonly caused by deletion of a segment of the maternally inherited chromosome 15q11-q13 region, which includes GABA_A_ receptor subunit genes, and results in disrupted GABA_A_ signaling (Egawa et al., 2008). The _MN_P35m component magnitude has also been shown to diminish in amplitude with increasing stimulation frequency, a feature similar to that observed with postsynaptic IPSPs (Deisz and Prince, 1989, Wikstrom et al., 1996). The _MN_P35m is also rapidly reduced at the beginning of a 10 Hz stimulus train (Huttunen and Lauronen, 2012) similar to the response from parvalbumin-containing inhibitory interneurons in primary somatosensory (SI) cortex (Beierlein et al., 2003, Reyes et al., 1998, Amiet et al., 2008). Unlike the _MN_N20m, the _MN_P35m has also been shown to be sensitive to on-going movement of the fingers, increasing with ipsilateral finger movement and decreasing with contralateral finger movement (Huttunen and Lauronen, 2012). Finally, unlike the tactile N30m, the tactile P50m is absent in newborns and begins to emerge after about 18 months (Nevalainen et al., 2014), possibly in relation to the known developmental change in GABA_A_ signaling from predominantly excitatory to inhibitory, due to changing intracellular chloride (Cl^−^) concentrations (Ganguly et al., 2001, Ben-Ari et al., 2012).

Given the evidence that these post-excitatory _MN_P35m or tactile P50m components may serve as indices of inhibitory signaling, it may be of interest to investigate these responses in clinical populations where cortical inhibition is thought to be impaired. Epilepsy, for example, is a diverse collection of neurological disorders characterized by sudden recurrent episodes of sensory disturbance, loss of consciousness, or convulsions, associated with abnormal synchronous electrical activity in the brain, and is generally thought to be the consequence of an imbalance between inhibitory and excitatory signaling (Bradford, 1995, Fritschy, 2008). Early demonstrations have shown that direct injection of GABA receptor antagonists (e.g., bicuculline, penicillin, pictotoxin) or glutamate receptor agonists (e.g., *N*-methyl-d-aspartate, NMDA) can trigger limbic motor seizures in rats and non-human primates (Iadarola and Gale, 1982, Piredda and Gale, 1985, Piredda et al., 1985, Mutani, 1986, Mutani et al., 1995). Since then, considerable research has taken place to identify medications that potentiate cortical GABA levels (e.g., vigabatrin and tiagabine). It should also be noted that, whereas there is general agreement that GABA and glutamate signaling are involved in seizure generation, the relationship is complex: in some cases, increasing GABA levels actually promotes seizures (Zhan and Nadler, 2009, Naylor et al., 2005, Scimemi et al., 2005, Klaassen et al., 2006), and medications acting on GABA and glutamate levels are far from perfectly effective in treating seizure disorders.

Another clinical population where impaired GABAergic signaling has been implicated is Autism Spectrum Disorder (ASD). ASD represents a distinct group of complex disorders of brain development characterized by difficulties in social interaction, verbal and nonverbal communication and repetitive behaviors (American_Psychiatric_Association, 2013). While clinically distinct from epilepsy, approximately 30% of children with ASD have a comorbid seizure disorder (Rossi et al., 1995, Tuchman and Rapin, 2002, Danielsson et al., 2005). Similar to epilepsy, a popular unifying theory for what causes ASD is an imbalance between excitation and inhibition in the brain, particularly in circuits governing sensory processes, memory, and social and emotional functions (Rubenstein and Merzenich, 2003). Various genetic and environmental factors may converge, in different combinations for each individual with ASD, thus resulting in aberrant excitation-inhibition (E/I) ratios (Rubenstein and Merzenich, 2003, Gao and Penzes, 2015). Indeed, epileptiform abnormalities on electroencephalograms and also seizures are known to occur at significantly higher rates in the most severely affected children with ASD (Gabis et al., 2005).

Evidence for reduced central nervous system (CNS) GABA levels has also been reported in unmedicated children with epilepsy as compared to age-matched controls (Rating et al., 1983). In vivo support for GABA deficiency in ASD continues to grow, with several replications documenting reduced magnetic resonance spectroscopy (MRS) evidence of GABA in motor, auditory, and somatosensory areas (Gaetz et al., 2014, Rojas et al., 2014, Puts et al., 2016). Thus, there is emerging consensus that shared developmental genetic, molecular and pathophysiological mechanisms exist and account for the common co-occurrence of ASD and epilepsy (Tuchman and Rapin, 2002). Recently, it has been shown that children with epilepsy report substantial problems in modulating their behavioral responses to sensory stimuli including both hypersensitivity to touch (tactile defensiveness) and tactile hyposensitivity (under-responsiveness) (van Campen et al., 2015). This collection of sensory processing disturbances is generally referred to as sensory modulation disorder (SMD). SMDs have been shown to perturb daily cognitive and behavioral functioning in 27% of otherwise typically developing (TD) children with epilepsy (van Campen et al., 2015). Together, these findings indicate that SMDs are a substantial yet under-recognized problem in childhood epilepsy and might also represent an important source of behavioral comorbidity with children and adults with ASD (van Campen et al., 2015).

Given the high prevalence of sensory dysfunction in ASD (> 70%) (Al-Heizan et al., 2015, Tomchek and Dunn, 2007, Adamson et al., 2006), previous neuroimaging studies have been conducted to assess whether the cortical somatosensory responses in ASD are atypical. For example, Marco et al. (2012) used MEG and reported that tactile P50m response amplitudes of the averaged evoked response are reduced in ASD, however these analyses were at the level of the MEG sensors (i.e. Global Field Power), and did not include source modeling (Marco et al., 2012). More recently, Khan et al. (2015) showed that a 50-Hz phase locking component of the vibrotactile response from primary somatosensory cortex was reduced in children with ASD (Khan et al., 2015). Additional research is needed to confirm whether ASD somatosensory responses differ in magnitude or latency from typical development (TD).

Somatosensory responses in patients with epilepsy have not been explored to the same degree as in ASD, and far less is known about whether the evoked response to tactile stimulation is atypical in children with epilepsy. However, it does appear that SEP latency and amplitude differences in epilepsy may depend on the type of epilepsy under evaluation. For example, using median nerve stimuli, Salas-Puig et al. (1992) compared SEPs between patients with juvenile myoclonic epilepsy (JME), idiopathic generalized epilepsy (IGE), and controls (Salas-Puig et al., 1992). The authors reported that the N19-P25 interval was significantly prolonged in the IGE group both as compared with the JME and control groups, but that no amplitude differences were noted between groups for the N19 component. However the P25 and N33 amplitudes were significantly higher in the JME group, including “giant SEPs” in 14% of JME patients (Salas-Puig et al., 1992). Another additional consideration is the use of anti-seizure medication in patients with epilepsy. While limited to case studies, there is some evidence that anti-epileptic medications such as phenytoin may increase SEP latency (Salas-Puig et al., 1992) whereas valproic acid may decrease SEP amplitudes (Kanazawa and Nagafuji, 1997).

The aim of the present study was to compare tactile P50m responses from a group of children with epilepsy and a group of children with ASD, with age matched TD controls. Given the evidence that 1) the tactile P50m response may represent GABA dependent post-excitatory inhibition and 2) GABA signaling is thought to be downregulated in both epilepsy and ASD, we hypothesized that separately, for both children with epilepsy and children with ASD, we would observe decreased SEF P50m response amplitudes to tactile stimulation of the digits. Whereas there is considerable support for auditory response latency differences of the N100m component between children with ASD and TD (Edgar et al., 2014, Gandal et al., 2010, Roberts et al., 2010), we have no direct evidence supporting a hypothesis of delayed latencies in the SEF tactile P50m peak response between ASD, TD and epilepsy groups.

## 2 Material and methods

### 2.1 Participants

The Children's Hospital of Philadelphia Institutional Review Board approved this study. Written parental informed consent and child assent was obtained from all participants.

ASD inclusion criteria: For this study, fifteen 8 to 12-year-old children with ASD (mean age 9.95 ± 1.23(SD) years; 3 female) were recruited from the Children's Hospital of Philadelphia (CHOP). Most children with ASD had a prior diagnosis, made by an expert clinician in CHOP's Regional Autism Center, according to DSM-IV or DSM-5 criteria. Some children had ASD educational classifications but no formal ASD medical diagnosis. Diagnostic and neurocognitive testing were performed to confirm ASD diagnosis, to ensure subjects met study inclusion/exclusion criteria, and to provide phenotypic characterization of the group. For children with diagnoses made by expert clinicians, given the extensive clinical evaluations upon which original diagnosis was made, an abbreviated diagnostic battery was used for confirmation and included standard diagnostic tools, including direct observation with the *Autism Diagnostic Observation Schedule-2nd Ed.* (ADOS-2) (Lord et al., 2000, Lord et al., 2012) and parent report on the *Social Communication Questionnaire* (*SCQ* (Rutter et al., 2003)). Dimensional symptom severity ratings were obtained by parent report on the *Social Responsiveness Scale-2nd Ed.* (*SRS-2* (Constantino and Gruber, 2005)) and by direct measurement using the ADOS-2 severity score metric (Gotham et al., 2009). The parent-completed *Autism Diagnostic Interview-Revised* (*ADI-R*) was administered for all participants who entered the study without a formal ASD diagnosis made by an expert clinician (e.g., ASD educational classification only) and for any child with a prior ASD diagnosis for whom a diagnostic discordance existed (e.g. a child who exceeded ADOS diagnostic cut-offs but was below SCQ and SRS-2 cut-offs). For final inclusion in the ASD group, children exceeded established cut-offs on the ADOS-2 and either the SCQ or SRS-2. Children 1 point below ADOS cut-offs were included in the ASD group if they exceeded cut-offs on at least two ASD parent-report questionnaires or on the ADI-R. Children without a prior diagnosis made by an expert clinician were required to exceed diagnostic cut-offs on both the ADOS-2 and ADI-R.

TD inclusion criteria: For this study, fifteen 8 to 12-year-old TD children (mean age 10.21 ± 1.61 (SD) years; 2 female) were recruited through pediatric practices of the CHOP primary care network. TD-specific inclusion criteria included scoring below the cut-off for ASD on the ADOS-2 as well as parent questionnaires, and performance above the 16th percentile on an index of language ability, the Clinical Evaluation of Language Fundamentals-4th or 5th Editions (CELF-4 or CELF-5) (Semel et al., 2003). Additional TD-specific exclusion/inclusion criteria included no history of psychiatric disorders and no developmental disorders or first-degree relatives with ASD. TD children were age and gender matched to the ASD group.

Additional inclusion/exclusion criteria for ASD and TD: All subjects were native English speakers with no known genetic syndromes, neurological disorders (e.g. epilepsy, cerebral palsy, traumatic brain injury (TBI)) or sensory impairments (somatosensory, hearing, visual). To rule out global cognitive delay, all participants also scored at or above the 5th percentile (SS > 70) on indices of nonverbal intelligence on the *Wechsler Intelligence Scale for Children–4th or 5th Editions* (*WISC-IV* or *WISC-V* (Wechsler, 2003)).

Epilepsy inclusion criteria: This study included seventeen 8–12 year old children (mean age 10.57 ± 1.72 (SD) years; 4 female) who visited the Children's Hospital of Philadelphia MEG center for clinical epilepsy (EPI) evaluation and consented to have their data used for research purposes. Exclusion criteria included atypical morphology of the perirolandic regions, whether as a result of prior surgery, trauma, or abnormal development; a more diffuse structural brain abnormality, or focal epilepsy with seizure onset zone localized to primary motor cortex (based on semiology and EEG recordings). Participants were selected to age and gender match the ASD patient group. Clinical data collected from the electronic medical record included a history of comorbid ASD, epilepsy characteristics, and medications at the time of imaging. None of the epilepsy patients had a history of comorbid ASD.

### 2.2 MEG recordings

All MEG recordings were performed at the Lurie Family Foundations' MEG Imaging Center of the Department of Radiology at the Children's Hospital of Philadelphia in a magnetically shielded room using a whole-cortex 275-channel MEG system (VSM MedTech Inc., Coquitlam, BC).

Three head-position indicator coils were attached to the scalp to provide continuous specification of the position and orientation of the MEG sensors relative to the head. Foam wedges were inserted between the side of each participant's head and the inside of the dewar to ensure immobility. To identify eye-blink activity, an electrooculogram (EOG) was collected. Electrodes were also applied over the left and right clavicles for electrocardiogram (ECG) recording. EOG/ECG artifacts were manually rejected per trial off-line. All recorded signals (EOG, ECG, and MEG) were digitized at 1200 Hz with 3rd order gradiometer environmental noise reduction applied to the MEG data. All participants were recorded in the supine position with eyes open.

Brain MRI images were obtained for each subject on a 3.0 Tesla Siemens Verio (TM) scanner using a 32-channel receive only head RF coil. For each participant we obtained a 3D Magnetization-Prepared Rapid Acquisition Gradient-Echo (MPRAGE) scan in an axial orientation, with field of view = 256 × 256 × 192 and matrix = 256 × 256 × 192 to yield 1 mm isotropic voxel resolution (TR/TE = 1900/2.87 ms; inversion time = 1100 ms; flip angle = 9 degrees).

### 2.3 Somatosensory stimuli

Somatosensory stimuli were presented to left and right index fingers separately using pneumatic pulses of compressed air delivered via clip-on balloon diaphragms. A pressure level of (30 p.s.i.) was optimized (from our extensive prior clinical pre-surgical mapping work) to achieve non-painful stimulation and a robust brain response from post-central somatosensory cortex. Stimulus duration was 35 ms, accommodating mechanical diaphragm elasticity and air-flow dispersion along the air tube from the compressed air source. The interstimulus interval (ISI) was jittered between 0.5 and 0.7 s. Data were collected in epochs of 0.4 s (− 0.1 to 0.3 s) for a total of 500 trials. These parameters were previously optimized from our extensive work with clinical pediatric populations (Roberts et al., 1995a, Roberts et al., 1995b, Schwartz et al., 2009). The average head position coordinates were calculated for each subject and used as a reference to identify and reject any trial with head motion in excess of 1 cm from the average head position. Somatosensory responses were averaged and then filtered between 1 and 40 Hz and the DC offset was removed using the pre-trigger 100 ms time period. Following this procedure, no differences in head motion were observed between groups, and no group differences existed in the number of trials per average (paired *t*-tests all > p = 0.05).

### 2.4 Equivalent current dipole (ECD) analysis

**Fig. 1:**
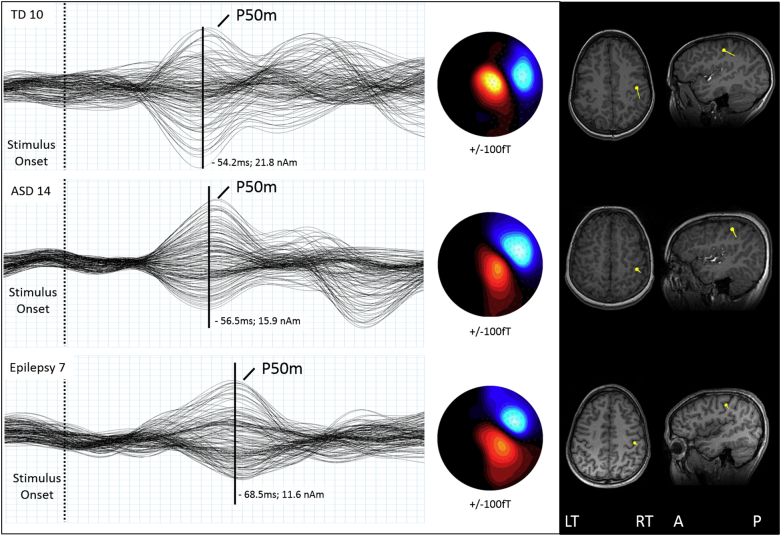
MEG averaged response waveforms from left index finger stimulation for three representative subjects (TD, ASD, and Epilepsy) are shown. Left: Vertical black line denotes tactile P50m peak and is scaled to the height of the TD response. Middle: tactile P50m field topography shows the dipolar field patterns for each tactile P50m peak response. Right: Coregistered ECD dipole locations for each tactile P50m responses are localized to contralateral primary somatosensory cortex for each subject. Note the larger amplitude in TD and the delayed response in epilepsy.

A tactile P50m response was observed in the evoked response average over contralateral somatosensory cortex for each stimulated finger. A spherical conductor model was manually fit to the inner skull surface of each subject's MRI. A single dipole model was fit to the time point of maximum field reversal (peak root mean square (RMS)) for the tactile P50m using a least-squares minimization algorithm and the resulting ECD was then coregistered on the subject's T1-weighted (3D MP-RAGE) MRI images. Dipole peak latency, location in cartesian coordinates (x, y, z), orientation, moment (nAm), and residual variance (R.V. %) were recorded separately for each subject for both (right and left) index fingers. Sensor montage included 143 sensors for left SEF and 143 sensors for right SEF which were selected to cover the maximum and minimum field topographies of the hemisphere contralateral to the hand of stimulation for all subjects. This approach was generally preferred to using a strictly left or right hemisphere sensor montage as SEF field maxima/minima may (depending on head size and position within the MEG dewar) occasionally cross the midline while still reducing the influence from known ipsilateral somatosensory responses (Korvenoja et al., 1995). Responses from three representative subjects are shown in Fig. 1.

Independently for both dipole moment and latency, we assessed effects of diagnostic group using a linear mixed model (LMM) with subject as a random effect and group and hemisphere as fixed effects and age as a covariate (a full factorial design was employed, including both 2- and 3-way interactions between group, hemisphere and age).

To ensure effects determined using the tests above were not secondary to systematic source localization or orientation biases, we conducted post-hoc assessment of effect of group on any of the three coordinates of source localization, or of source orientation in analogous independent LMM's. Note, for the y-coordinate of source localization (left-right axis), we conducted separate LMM's for left and right digit stimulation.

## 3 Results

### 3.1 Participant characteristics

The enrolled cohorts consisted of 15 TD, 15 ASD, and 17 EPI participants. Clinical characteristics are summarized in Table 1, Table 2, Table 3 respectively. No ASD participant had a parent report or clinical diagnosis of seizures. None of the EPI participants met diagnostic criteria for ASD, nor had a pre-existing ASD diagnosis. Number of antiepileptic medications ranged from none to three. Seven Epilepsy patients had visible MRI lesions and 10 had no structural lesions and epilepsy of unknown etiology (see Table 3).

**Table 1:**
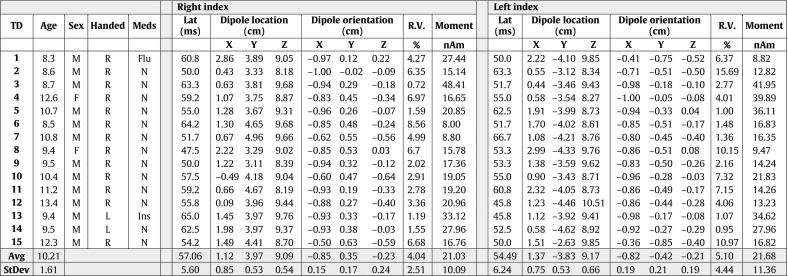
TD Demographics and ECD table.

**Table 2:**
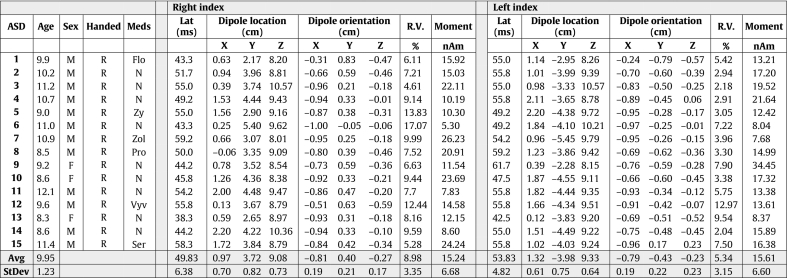
ASD Demographics and ECD table.

**Table 3:**
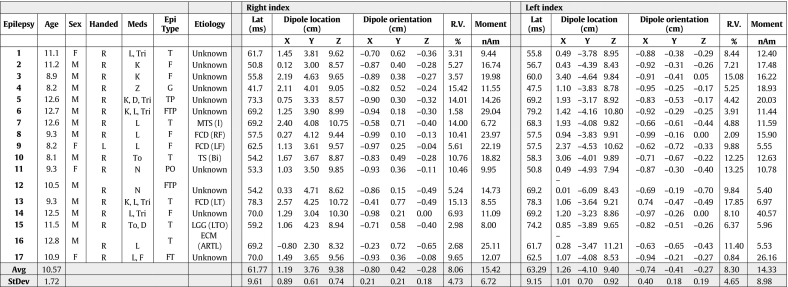
Epilepsy demographics and ECD table.

#### 3.1.1 Dipole moment

A linear mixed model (LMM) was performed on dipole moment, with subject as a random effect and group and hemisphere as fixed effects and age as a covariate (a full factorial design was employed, including both 2- and 3-way interactions between group, hemisphere and age). The choice to use hemisphere as a covariate stemmed from previous published literature showing decreased amplitude in the P50m response in ASD for left hemisphere only (Marco et al., 2012). We observed a significant overall effect of group F(2,41) = 4.21, p < 0.05. Independent post-hoc pair-wise comparisons for dipole moment showed marginal means (age 10.26 years) were significantly different comparing TD (21.3 ± 7.0nAm(SD)) and epilepsy (14.7 ± 6.6nAm), p = 0.014, as well as for TD vs ASD (15.4 ± 7.4nAm), p = 0.027. There was no significant effect of hemisphere (left digit tapped, right hemisphere response: 17.1 ± 5.0nAm; right digit tapped, left hemisphere response: 17.2 ± 5.0nAm, F(1,41) = 0.002, p > 0.9) or age, nor were there any significant group X age or group X hemisphere or group X hemisphere X age interactions (all p's > 0.05).

**Fig. 2:**
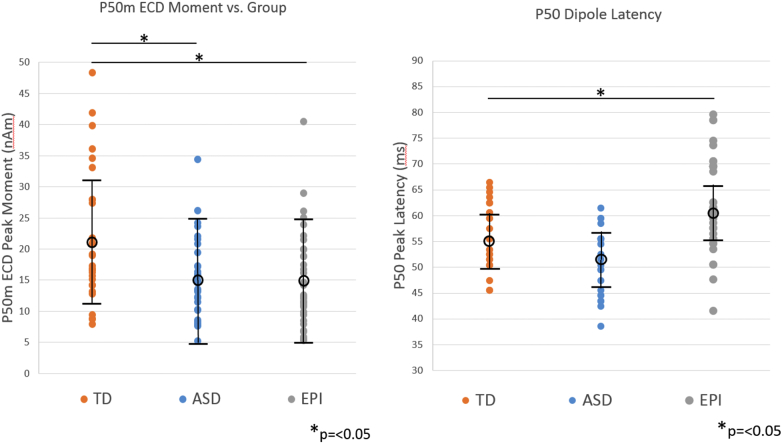
Left panel: Group mean and standard deviations for dipole moment reveal a significant overall effect of group F(2,41) = 4.21, p < 0.05. Independent post-hoc pair wise comparisons showed means were significantly different TD (21.3 ± 7.0nAm(SD)) vs epilepsy (14.7 ± 6.6nAm), p < 0.02 and TD vs ASD (15.4 ± 7.4nAm). Right panel: Group mean and standard deviations for dipole peak latency: Significant overall effect of group F(2,41) = 12.0, p < 0.05. Post-hoc pair wise comparisons showed this to be driven by a latency delay of ~ 6 ms in the epilepsy group: means were significantly different TD (55.8 ± 5.4 ms) vs epilepsy (61.6 ms ± 5.0 ms), p < 0.02, but not significant for TD vs ASD (52.4 ± 5.4 ms), p = 0.9.

Furthermore, within the epilepsy cohort, there was no significant difference in lesional vs non-lesional cases (considering the potential main effect of “lesion” in an equivalent LMM applied to the epilepsy cohort) with marginal means (age 10.57 years): non-lesional 15.0 ± 6.2nAm, lesional 17.4 ± 13.9nAm, F(1,25) = 0.353, p = 0.558 (See Fig. 2 left panel).

#### 3.1.2 Dipole peak latency

Similarly, a linear mixed model was performed on dipole peak latency, with subject as a random effect and group and hemisphere as fixed effects and age as a covariate. We observed a significant overall effect of group F(2,41) = 12.0, p < 0.05. Post-hoc pair wise comparisons for dipole latency showed this to be driven by a latency delay of ~ 6 ms in the epilepsy group: marginal means (age 10.26 years) were significantly different comparing TD (55.8 ± 5.4 ms) and epilepsy (61.6 ms ± 5.0 ms), p = 0.01, and not significant for TD vs ASD (52.4 ± 5.4 ms), p > 0.05. There was no significant effect of hemisphere (left digit tapped, right hemisphere response: 57.0 ± 3.9 ms; right digit tapped, left hemisphere response: 56.2 ± 3.9 ms, F(1,41) = 0.463, p = 0.5) or age, nor were there any significant group X age or group X hemisphere or group X hemisphere X age interactions (all p's > 0.05) (See Fig. 2 right panel).

Furthermore, within the epilepsy cohort, there was no significant difference in lesional vs non-lesional cases (considering the potential main effect of “lesion” in an equivalent LMM applied to the epilepsy cohort) with marginal means (age 10.57 years): non-lesional 61.8 ± 7.4 ms, lesional 59.8 ± 12.8 ms, F(1,25) = 0.407, p = 0.533, (Fig. 2).

Post-hoc assessment of effect of group on any of the three coordinates of source localization, or of source orientation in independent LMM's showed no significant effect of Group on any source localization parameter (all p's > 0.4), eliminating the possible influence of source localization bias on the above estimates of moment and latency effects.

## 4 Discussion

The aim of the current study was to assess whether the somatosensory tactile P50m response in children with ASD and children with epilepsy was reduced compared to TD controls. We hypothesized that the magnitude of the tactile P50m somatosensory response would be reduced in both patient groups, possibly due to reduced GABAergic signaling as has been implicated in a variety of previous animal models and in vivo human MRS studies. We observed significant (~ 25%) decreases in tactile P50m dipole moment values, both for children with epilepsy and for children with ASD. In addition, the latency of the tactile P50m peak was observed to be not different between TD and ASD groups but was significantly delayed in children with epilepsy by ~ 6 ms.

The observed decrease in dipole moment in both the ASD and epilepsy patient groups is consistent with the model of reduced GABAergic signaling in the brains of these clinical populations. Direct support for downregulation of somatosensory GABA signaling in children with ASD comes from Puts et al. (2016) who reported significantly reduced GABA concentration from somatosensory ROIs in a group of children with ASD (Puts et al., 2016). Interestingly Puts et al. (2016) show GABA levels reduced by a similar proportion as the SEF moments reported herein. Puts et al. (2016) also showed that GABA levels in healthy children were observed to correlate with measures of tactile performance, but in typically developing children only and not in children with ASD (Puts et al., 2016), hinting at a specific somatosensory GABA/behavioral impairment in ASD. Using similar MRS methods, patients with epilepsy have also been shown to have significantly reduced in vivo GABA (Petroff et al., 1996, Wang et al., 2003) although evidence of a direct behavioral correlate of GABA downregulation in the somatosensory domain in epilepsy remains lacking.

Presently, there are only two published findings assessing neuromagnetic responses to tactile stimuli in ASD. Marco et al. (2012) employed a somatosensory oddball task where somatosensory stimuli were presented at slow (ISI 1.32–2.64 s), and fast (ISI 0.33 s) as well as a standard oddball task in a group of ASD children and observed reduced amplitudes in peak RMS (30–70 ms windows) for the slow ISI condition only (right hemisphere only) and with no latency differences between groups (Marco et al., 2012). Our current findings using an intermediate rate of stimulus presentation (ISI of 0.5–0.7 s) are in general agreement with Marco et al. (2012) and extend these findings to include source localized ECD responses; however, we did not observe hemispheric asymmetries in the reduced tactile P50m response. Our results are also consistent with Khan et al. (2015) who reported a significant decrease in 50 Hz phase locking component of the vibrotactile responses in children with ASD (van Campen et al., 2015). Our findings are also generally consistent with the model of a global reduction in sensory processing as prior studies have shown decreased cortical responses to both visual and auditory responses in ASD. For example, a recent study measuring transient visual evoked potentials in children with ASD reported reduced amplitudes in both the early excitatory P60-N75 potentials as well as the post-excitatory N75-P100 potentials with no evidence of a latency delay (Siper et al., 2016). In addition, Wilson et al. (2007) previously reported decreased auditory steady-state responses in children and adolescents with ASD (Wilson et al., 2007). While these data generally support a global deficit in cortical response amplitudes across all sensory modalities in children with ASD they are also distinct from the well-known finding of a delayed N100m auditory response consistently observed in these children (Edgar et al., 2014, Gandal et al., 2010, Roberts et al., 2010).

We observed an ~ 6 ms delay in tactile P50m somatosensory response in our epilepsy participants which has not been reported previously. A prolonged N19-P25 interval has, however been reported in patients with idiopathic generalized epilepsy (IGE) using median nerve stimulation (Salas-Puig et al., 1992). Both visual (Verleger et al., 1997) and auditory delays (Tome et al., 2014) have been observed in the evoked responses of adults and children with epilepsy. We speculate that the influence of anti-epileptic medications may be a contributing factor in promoting the tactile P50m latency delay in our epilepsy group (see Table 3). However, evidence does exist where median nerve P25 somatosensory response amplitude (but not latency) was reported to be reduced following valproate administration (Kanazawa and Nagafuji, 1997). In the current study, only 2 of our patients were taking valproate at the time of study and thus did not likely bias the observed group effects of decreased moment and increased latency in the epilepsy group (see Table 3).

There are, however, additional factors that could also have contributed to the observed latency delay and reduced ECD moment in our epilepsy patients. One such factor is the potential reorganization of sensorimotor representations that have been demonstrated on a cellular and molecular level in animal models following experimentally induced febrile seizures. As little as a single infantile seizure was demonstrated to alter the expression of several proteins (Reid et al., 2011) and cortical excitability into adulthood (Reid et al., 2011, Isaeva et al., 2009). Human studies have also shown cortical excitability to be affected in patients with temporal lobe epilepsy, with inter-ictal responses to peripheral somatosensory stimulation reduced compared to healthy control subjects (Hsu et al., 2015). As such, disruptions in the sensorimotor functional network due to brain reorganization as a result of prolonged seizures could potentially be impacting sensorimotor cortical responses to the peripheral stimulation provided to our group of patients. Individuals with malformations of cortical development in sensorimotor cortical areas have been shown to have MEG responses to somatosensory stimulation mapped to anatomic locations outside of the expected regions (Burneo et al., 2004), which could result in delayed response latencies, although likely less of an issue in our particular patient group as none of them had gross malformations in primary sensorimotor cortex, nor significantly atypical source localizations to somatosensory stimulation. Neoplasms have also been shown to alter early somatosensory responses to electrical median nerve stimulation as measured by MEG, possibly due to alterations in glial cell activity in neoplastic cells (Chang et al., 2013). While residual postoperative neoplasm in patients may in principle contribute to the delayed tactile P50m response, only one of our epilepsy patients had a non-postoperative neoplasm (glioma). Although none of our patients had known significant metabolic derangements, hepatic encephalopathy has also been shown to delay the cortical response to electric median nerve stimulation (May et al., 2014); mild or subclinical imbalances, such as those seen in patients experiencing seizures, could also have contributed to this finding in our epilepsy group.

As in any neurophysiologic studies of patients with epilepsy, disentangling the effects of the underlying disorder from the effects of anti-seizure medications with widely varying mechanisms of action is difficult. The sample size of the cohort in this study is under powered to make comparisons between medications or mechanism of action categories. An additional complicating factor may also be the natural fluctuations in cortical excitability related to peri-ictal or interictal states in patients with epilepsy. Future studies within more homogeneous epilepsy populations may enable further delineation of the many factors potentially contributing to the findings observed here. Similarly, the lack of statistical power reduces our ability to make inferences about differential effects in lesional vs non-lesional cases. In the analyses performed no significant differences were found in moment or latency, but this should be interpreted with caution given the rather few lesional cases (see Table 3).

One novel aspect of the current study was the observation of significantly reduced P50m response amplitudes in both ASD and epilepsy, not inconsistent with comorbidity of reduced inhibitory signaling in both groups. The leading theory of shared etiology of Epilepsy and leading theories of ASD point to an imbalance of excitation and inhibition at the synapse (Fritschy, 2008, Rubenstein and Merzenich, 2003), and some validity to this shared etiology hypothesis is offered by the comorbidity of diagnoses. The earliest manuscript describing ASD includes a discussion regarding the association of ASD with epilepsy (Kanner, 1943) and greater comorbidity with epilepsy than that expected by chance alone (Tuchman and Rapin, 2002, Matsuo et al., 2010, Tuchman and Cuccaro, 2011, Tuchman et al., 2013). Recent estimates suggest that the rate of epilepsy in adolescents and adults with ASD is 20%, and the rate of ASD in epilepsy is 8% (Kohane et al., 2012, Rai et al., 2012). These estimates are far greater than the prevalence of epilepsy or ASD in the general population (1 in 68 for ASD (Bhat et al., 2014, Baio, 2014), and 5–10 in 1000 for Epilepsy (Sander, 2003)) respectively. A key factor in these rates appears to be the importance of intellectual disability in modulating the co-morbid relationship between autism and epilepsy (Spence and Schneider, 2009, Berg and Plioplys, 2012). In children with ASD, having co-occurring epilepsy has been associated with a higher rate of cognitive deficits, and frontal lobe epileptiform activity (Matsuo et al., 2011) when compared with children with ASD alone. However, studies have also indicated that even in cases with normal IQ there is evidence that autism itself is associated with an increased risk of epilepsy (Amiet et al., 2008, Olsson et al., 1988, Elia et al., 1995).

To better identify shared mechanisms between epilepsy and ASD, it is becoming increasingly common to investigate genetic disorders for which both epilepsy and ASD commonly occur, such as Fragile X, Rett and Angelman syndrome, and tuberous sclerosis (TS), (Brooks-Kayal, 2010, Trillingsgaard and O.S., 2004, Curatolo et al., 2015) (see Buckley and Holmes (2016) for a recent review). For example, in children with TS typically due to a mutation of Tsc1 gene (encoding for the protein hamartin) or Tsc2 gene (encoding for the protein tuberin) (Curatolo et al., 2015), early seizure onset is associated with a high risk for ASD (Numis et al., 2011, van Eeghen et al., 2012). Not only are patients with TS more likely to have ASD (~ 40%) (Numis et al., 2011), these rates can vary depending on whether children start anti-epileptic medications early (9%) or are treated later on in childhood (52%) (Cusmai et al., 2011, Bombardieri et al., 2009). Thus it appears that treatment with vigabatrin – (a GABA agonist that inhibits the breakdown of γ-aminobutyric acid) acts to restore E/I balance and prevent seizures. However, if left untreated during early neurodevelopment, the likelihood of ASD increases substantially in children with TS (Cusmai et al., 2011, Bombardieri et al., 2009).

Of course, there are multiple genetic and environmental causes for both ASD and epilepsy, but both have been conceptualized as disorders of aberrant E/I ratio via reduced inhibitory signaling which may possibly be explained by the same early insult. For example, a specific genetic mutation may impair the promotion of inhibitory signaling in the cortex, and thus disrupt the E/I balance. This disruption may then promote early life seizure activity resulting in seizures or devastating impairments in social communication and behavior, or both. The current study lends additional support for the theory that GABA signaling is downregulated both in the brains of children with ASD and in children with epilepsy. To better understand the relationship between GABA, cortical signaling and behavior, further work is needed to replicate our current findings in clearly stratified groups (EPI only; ASD only) as well as a comorbid group (ASD + EPI) and directly relate them to both in vivo measures of GABA via, e.g. MRS spectroscopy and psychophysical assessments of somatosensory function, as well as paired behavioral measures.

## References

[bb0005] Adamson A., O'Hare A., Graham C. (2006). Impairments in sensory modulation in children with autistic spectrum disorder. Br. J. Occup. Ther..

[bb0010] Al-Heizan M.O. (2015). Sensory processing dysfunction among Saudi children with and without autism. J. Phys. Ther. Sci..

[bb0015] Allison T. (1989). Human cortical potentials evoked by stimulation of the median nerve. II. Cytoarchitectonic areas generating long-latency activity. J. Neurophysiol..

[bb0020] Allison T. (1991). Potentials evoked in human and monkey cerebral cortex by stimulation of the median nerve. A review of scalp and intracranial recordings. Brain.

[bb0025] American_Psychiatric_Association (2013). Diagnostic and Statistical Manual of Mental Disorders.

[bb0030] Amiet C. (2008). Epilepsy in autism is associated with intellectual disability and gender: evidence from a meta-analysis. Biol. Psychiatry.

[bb0035] Anziska B.J., Cracco R.Q. (1983). Short-latency somatosensory evoked potentials to median nerve stimulation in patients with diffuse neurologic disease. Neurology.

[bb0040] Baio J. (2014). Prevalence of Autism Spectrum Disorder Among Children Aged 8 Years—11 Sites Autism and Developmental Disabilities Monitoring Network.

[bb0045] Baumgartner C. (1991). Neuromagnetic investigation of somatotopy of human hand somatosensory cortex. Exp. Brain Res..

[bb0050] Baumgartner C. (1993). Somatotopy of human hand somatosensory cortex as studied in scalp EEG. Electroencephalogr. Clin. Neurophysiol..

[bb0055] Beierlein M., Gibson J.R., Connors B.W. (2003). Two dynamically distinct inhibitory networks in layer 4 of the neocortex. J. Neurophysiol..

[bb0060] Ben-Ari Y. (2012). Refuting the challenges of the developmental shift of polarity of GABA actions: GABA more exciting than ever!. Front. Cell. Neurosci..

[bb0065] Berg A.T., Plioplys S. (2012). Epilepsy and autism: is there a special relationship?. Epilepsy Behav..

[bb0070] Bhat S. (2014). Autism: cause factors, early diagnosis and therapies. Rev. Neurosci..

[bb0075] Bombardieri R. (2009). Early control of seizures improves long-term outcome in children with tuberous sclerosis complex. Eur. J. Paediatr. Neurol..

[bb0080] Bourguignon M. (2014). Corticokinematic coherence mainly reflects movement-induced proprioceptive feedback. NeuroImage.

[bb0085] Bradford H.F. (1995). Glutamate, GABA and epilepsy. Prog. Neurobiol..

[bb0090] Brooks-Kayal A. (2010). Epilepsy and autism spectrum disorders: are there common developmental mechanisms?. Brain Dev..

[bb0095] Buckley A.W., Holmes G.L. (2016). Epilepsy and autism. Cold Spring Harb. Perspect. Med..

[bb0100] Burneo J.G. (2004). Cortical reorganization in malformations of cortical development: a magnetoencephalographic study. Neurology.

[bb0105] van Campen J.S. (2015). Sensory modulation disorders in childhood epilepsy. J. Neurodev. Disord..

[bb0110] Chang W.S. (2013). Decreased inhibitory neuronal activity in patients with frontal lobe brain tumors with seizure presentation: preliminary study using magnetoencephalography. Acta Neurochir..

[bb0115] Cheyne D. (2003). Neuromagnetic imaging of cortical oscillations accompanying tactile stimulation. Brain Res. Cogn. Brain Res..

[bb0120] Constantino J.N., Gruber C.P. (2005). Social Responsiveness Scale.

[bb0125] Cracco R.Q. (1982). Short-latency somatosensory evoked potentials to median and peroneal nerve stimulation: studies in normal subjects and patients with neurologic disease. Ann. N. Y. Acad. Sci..

[bb0130] Curatolo P., Moavero R., de Vries P.J. (2015). Neurological and neuropsychiatric aspects of tuberous sclerosis complex. Lancet Neurol..

[bb0135] Cusmai R. (2011). Long-term neurological outcome in children with early-onset epilepsy associated with tuberous sclerosis. Epilepsy Behav..

[bb0140] Danielsson S. (2005). Epilepsy in young adults with autism: a prospective population-based follow-up study of 120 individuals diagnosed in childhood. Epilepsia.

[bb0145] Deisz R.A., Prince D.A. (1989). Frequency-dependent depression of inhibition in guinea-pig neocortex in vitro by GABAB receptor feed-back on GABA release. J. Physiol..

[bb0150] Edgar J.C. (2014). Missing and delayed auditory responses in young and older children with autism spectrum disorders. Front. Hum. Neurosci..

[bb0155] van Eeghen A.M. (2012). Understanding relationships between autism, intelligence, and epilepsy: a cross-disorder approach. Dev. Med. Child Neurol..

[bb0160] Egawa K. (2008). Aberrant somatosensory-evoked responses imply GABAergic dysfunction in Angelman syndrome. NeuroImage.

[bb0165] Elia M. (1995). Clinical and neurophysiological aspects of epilepsy in subjects with autism and mental retardation. Am. J. Ment. Retard..

[bb0170] Forss N., Salmelin R., Hari R. (1994). Comparison of somatosensory evoked fields to airpuff and electric stimuli. Electroencephalogr. Clin. Neurophysiol..

[bb0175] Fritschy J.M. (2008). Epilepsy, E/I balance and GABA(A) receptor plasticity. Front. Mol. Neurosci..

[bb0180] Gabis L., Pomeroy J., Andriola M.R. (2005). Autism and epilepsy: cause, consequence, comorbidity, or coincidence?. Epilepsy Behav..

[bb0185] Gaetz W., Cheyne D. (2006). Localization of sensorimotor cortical rhythms induced by tactile stimulation using spatially filtered MEG. NeuroImage.

[bb0190] Gaetz W. (2014). GABA estimation in the brains of children on the autism spectrum: measurement precision and regional cortical variation. NeuroImage.

[bb0195] Gandal M.J. (2010). Validating gamma oscillations and delayed auditory responses as translational biomarkers of autism. Biol. Psychiatry.

[bb0200] Ganguly K. (2001). GABA itself promotes the developmental switch of neuronal GABAergic responses from excitation to inhibition. Cell.

[bb0205] Gao R., Penzes P. (2015). Common mechanisms of excitatory and inhibitory imbalance in schizophrenia and autism spectrum disorders. Curr. Mol. Med..

[bb0210] Gotham K., Pickles A., Lord C. (2009). Standardizing ADOS scores for a measure of severity in autism spectrum disorders. J. Autism Dev. Disord..

[bb0215] Hari R., Forss N. (1999). Magnetoencephalography in the study of human somatosensory cortical processing. Philos. Trans. R. Soc. Lond. Ser. B Biol. Sci..

[bb0220] Hari R. (1984). Somatosensory evoked cerebral magnetic fields from SI and SII in man. Electroencephalogr. Clin. Neurophysiol..

[bb0225] Hsu W.Y. (2015). Widespread inter-ictal excitability changes in patients with temporal lobe epilepsy: a TMS/MEG study. Epilepsy Res..

[bb0230] Huttunen J., Lauronen L. (2012). Intracortical modulation of somatosensory evoked fields during movement: evidence for selective suppression of postsynaptic inhibition. Brain Res..

[bb0235] Iadarola M.J., Gale K. (1982). Substantia nigra: site of anticonvulsant activity mediated by gamma-aminobutyric acid. Science.

[bb0240] Ikeda H., Wang Y., Okada Y.C. (2005). Origins of the somatic N20 and high-frequency oscillations evoked by trigeminal stimulation in the piglets. Clin. Neurophysiol..

[bb0245] Inoue K. (2013). Somatosensory mechanical response and digit somatotopy within cortical areas of the postcentral gyrus in humans: an MEG study. Hum. Brain Mapp..

[bb0250] Isaeva E. (2009). Long-term suppression of GABAergic activity by neonatal seizures in rat somatosensory cortex. Epilepsy Res..

[bb0255] Jamali S., Ross B. (2013). Somatotopic finger mapping using MEG: toward an optimal stimulation paradigm. Clin. Neurophysiol..

[bb0260] Kakigi R. (2000). The somatosensory evoked magnetic fields. Prog. Neurobiol..

[bb0265] Kanazawa O., Nagafuji H. (1997). Valproate lowered the amplitude of visual and somatosensory evoked potentials in two cases of untreated juvenile myoclonic epilepsy. Psychiatry Clin. Neurosci..

[bb0270] Kanner L. (1943). Autistic disturbances of affective contact. Nerv. Child.

[bb0275] Khan S. (2015). Somatosensory cortex functional connectivity abnormalities in autism show opposite trends, depending on direction and spatial scale. Brain.

[bb0280] Klaassen A. (2006). Seizures and enhanced cortical GABAergic inhibition in two mouse models of human autosomal dominant nocturnal frontal lobe epilepsy. Proc. Natl. Acad. Sci. U. S. A..

[bb0285] Kohane I.S. (2012). The co-morbidity burden of children and young adults with autism spectrum disorders. PLoS One.

[bb0290] Korvenoja A. (1995). Activation of ipsilateral primary sensorimotor cortex by median nerve stimulation. Neuroreport.

[bb0295] Lin Y.Y. (2005). Differential generators for N20m and P35m responses to median nerve stimulation. NeuroImage.

[bb0300] Lord C. (2000). The autism diagnostic observation schedule-generic: a standard measure of social and communication deficits associated with the spectrum of autism. J. Autism Dev. Disord..

[bb0305] Lord C. (2012). Autism Diagnostic Observation Schedule (ADOS-2).

[bb0310] Marco E.J. (2012). Children with autism show reduced somatosensory response: an MEG study. Autism Res..

[bb0315] Matsumiya Y., Mostofsky D.I. (1972). Somatosensory evoked responses elicited by corneal and nostril air puff stimulation. Electroencephalogr. Clin. Neurophysiol..

[bb0320] Matsuo M. (2010). Frequent association of autism spectrum disorder in patients with childhood onset epilepsy. Brain Dev..

[bb0325] Matsuo M. (2011). Characterization of childhood-onset complex partial seizures associated with autism spectrum disorder. Epilepsy Behav..

[bb0330] Mauguiere F. (1997). Activation of a distributed somatosensory cortical network in the human brain. A dipole modelling study of magnetic fields evoked by median nerve stimulation. Part I: location and activation timing of SEF sources. Electroencephalogr. Clin. Neurophysiol..

[bb0335] May E.S. (2014). Hepatic encephalopathy is associated with slowed and delayed stimulus-associated somatosensory alpha activity. Clin. Neurophysiol..

[bb0340] Mutani R. (1986). Neurophysiological mechanisms underlying epileptogenesis. Funct. Neurol..

[bb0345] Mutani R. (1995). Antiepileptic drugs and mechanisms of epileptogenesis. A review. Ital. J. Neurol. Sci..

[bb0350] Nangini C. (2006). Magnetoencephalographic study of vibrotactile evoked transient and steady-state responses in human somatosensory cortex. NeuroImage.

[bb0355] Naylor D.E., Liu H., Wasterlain C.G. (2005). Trafficking of GABA(A) receptors, loss of inhibition, and a mechanism for pharmacoresistance in status epilepticus. J. Neurosci..

[bb0360] Nevalainen P., Lauronen L., Pihko E. (2014). Development of human somatosensory cortical functions - what have we learned from magnetoencephalography: a review. Front. Hum. Neurosci..

[bb0365] Numis A.L. (2011). Identification of risk factors for autism spectrum disorders in tuberous sclerosis complex. Neurology.

[bb0370] Olsson I., Steffenburg S., Gillberg C. (1988). Epilepsy in autism and autisticlike conditions. A population-based study. Arch. Neurol..

[bb0375] Petroff O.A. (1996). Low brain GABA level is associated with poor seizure control. Ann. Neurol..

[bb0380] Pihko E. (2009). Maturation of somatosensory cortical processing from birth to adulthood revealed by magnetoencephalography. Clin. Neurophysiol..

[bb0385] Piredda S., Gale K. (1985). A crucial epileptogenic site in the deep prepiriform cortex. Nature.

[bb0390] Piredda S., Lim C.R., Gale K. (1985). Intracerebral site of convulsant action of bicuculline. Life Sci..

[bb0395] Puts N.A. (2016). Reduced GABA and altered somatosensory function in children with autism spectrum disorder. Autism Res..

[bb0400] Rai D. (2012). Epilepsy and psychiatric comorbidity: a nationally representative population-based study. Epilepsia.

[bb0405] Rating D., Siemes H., Loscher W. (1983). Low CSF GABA concentration in children with febrile convulsions, untreated epilepsy, and meningitis. J. Neurol..

[bb0410] Reid A.Y., Pittman Q.J., Teskey G.C. (2011). A prolonged experimental febrile seizure results in motor map reorganization in adulthood. Neurobiol. Dis..

[bb0415] Reyes A. (1998). Target-cell-specific facilitation and depression in neocortical circuits. Nat. Neurosci..

[bb0420] Roberts T., Rowley H., Kucharczyk J. (1995). Applications of magnetic source imaging to presurgical brain mapping. Neuroimaging Clin. N. Am..

[bb0425] Roberts T.P. (1995). Correlation of functional magnetic source imaging with intraoperative cortical stimulation in neurosurgical patients. J. Image Guid. Surg..

[bb0430] Roberts T.P. (2010). MEG detection of delayed auditory evoked responses in autism spectrum disorders: towards an imaging biomarker for autism. Autism Res..

[bb0435] Rojas D.C. (2014). Decreased left perisylvian GABA concentration in children with autism and unaffected siblings. NeuroImage.

[bb0440] Rossi P.G. (1995). EEG features and epilepsy in patients with autism. Brain Dev..

[bb0445] Rubenstein J.L., Merzenich M.M. (2003). Model of autism: increased ratio of excitation/inhibition in key neural systems. Genes Brain Behav..

[bb0450] Rutter M., Le Couteur A., Lord C. (2003). Autism Diagnostic Interview—Revised.

[bb0455] Salas-Puig J. (1992). Somatosensory evoked potentials in juvenile myoclonic epilepsy. Epilepsia.

[bb0460] Sander J.W. (2003). The epidemiology of epilepsy revisited. Curr. Opin. Neurol..

[bb0465] Schieppati M., Ducati A. (1984). Short-latency cortical potentials evoked by tactile air-jet stimulation of body and face in man. Electroencephalogr. Clin. Neurophysiol..

[bb0470] Schwartz E.S. (2009). Magnetoencephalography. Pediatr. Radiol..

[bb0475] Scimemi A. (2005). Multiple and plastic receptors mediate tonic GABAA receptor currents in the hippocampus. J. Neurosci..

[bb0480] Semel E., Wiig E.H., Secord W. (2003). Clinical Evaluation of Language Fundamentals-IV.

[bb0485] Seyal M., Mull B. (2002). Mechanisms of signal change during intraoperative somatosensory evoked potential monitoring of the spinal cord. J. Clin. Neurophysiol..

[bb0490] Sheth S.A. (2013). Factors affecting successful localization of the central sulcus using the somatosensory evoked potential phase reversal technique. Neurosurgery.

[bb0495] Siper P.M. (2016). Rapid and objective assessment of neural function in autism spectrum disorder using transient visual evoked potentials. PLoS One.

[bb0500] Spence S.J., Schneider M.T. (2009). The role of epilepsy and epileptiform EEGs in autism spectrum disorders. Pediatr. Res..

[bb0505] Tomchek S.D., Dunn W. (2007). Sensory processing in children with and without autism: a comparative study using the short sensory profile. Am. J. Occup. Ther..

[bb0510] Tome D. (2014). Auditory event-related potentials in children with benign epilepsy with centro-temporal spikes. Epilepsy Res..

[bb0515] Trillingsgaard A., O.S. J.R. (2004). Autism in Angelman syndrome: an exploration of comorbidity. Autism.

[bb0520] Tuchman R., Cuccaro M. (2011). Epilepsy and autism: neurodevelopmental perspective. Curr. Neurol. Neurosci. Rep..

[bb0525] Tuchman R., Rapin I. (2002). Epilepsy in autism. Lancet Neurol..

[bb0530] Tuchman R., Hirtz D., Mamounas L.A. (2013). NINDS epilepsy and autism spectrum disorders workshop report. Neurology.

[bb0535] Verleger R. (1997). Event-related potentials suggest slowing of brain processes in generalized epilepsy and alterations of visual processing in patients with partial seizures. Brain Res. Cogn. Brain Res..

[bb0540] Walsh J.C. (1982). Evoked potential changes in clinically definite multiple sclerosis: a two year follow up study. J. Neurol. Neurosurg. Psychiatry.

[bb0545] Wang Z.J. (2003). In vivo measurement of brain metabolites using two-dimensional double-quantum MR spectroscopy—exploration of GABA levels in a ketogenic diet. Magn. Reson. Med..

[bb0550] Wechsler D. (2003). Wechsler Intelligence Scale for Children - IV Integrated.

[bb0555] Wikstrom H. (1996). Effects of interstimulus interval on somatosensory evoked magnetic fields (SEFs): a hypothesis concerning SEF generation at the primary sensorimotor cortex. Electroencephalogr. Clin. Neurophysiol..

[bb0560] Wilson T.W. (2007). Children and adolescents with autism exhibit reduced MEG steady-state gamma responses. Biol. Psychiatry.

[bb0565] Wood C.C. (1985). Electrical sources in human somatosensory cortex: identification by combined magnetic and potential recordings. Science.

[bb0570] Xiang J. (1997). Somatosensory evoked magnetic fields following passive finger movement. Brain Res. Cogn. Brain Res..

[bb0575] Zhan R.Z., Nadler J.V. (2009). Enhanced tonic GABA current in normotopic and hilar ectopic dentate granule cells after pilocarpine-induced status epilepticus. J. Neurophysiol..

